# Lamotrigine and Fluoxetine Overdose Presenting With Severe Neuro-Cardiotoxicity: A Case Report

**DOI:** 10.7759/cureus.108026

**Published:** 2026-04-30

**Authors:** Sujeet Joshi, Asif Ahmed, Amit Sharma, Ravikant Verma, Prakash Singh

**Affiliations:** 1 Critical Care Medicine, Tata Main Hospital, Jamshedpur, IND

**Keywords:** acute kidney injury, anticonvulsant therapy, atrial fibrillation management, invasive mechanical ventilation, lamotrigine fluoxetine combination therapy, life threatening arrhythmia, refractory status epilepticus, subclinical aspiration pneumonia, treatment-resistant depression, vocal cord dysfunction

## Abstract

Lamotrigine and fluoxetine are commonly prescribed for the treatment of bipolar disorder and anxiety. Combination therapy is used in resistant bipolar depression to improve synergy and tolerability. Although most cases of lamotrigine overdose have mild toxicity, in the case of massive ingestion of more than 4 g associated with severe neurological and cardiac complications, leading to mortality, either due to severe central nervous system depression, refractory seizures, or due to cardiac arrhythmia. We describe the case of a 28‑year‑old woman with bipolar disorder who developed status epilepticus, atrial fibrillation, aspiration pneumonia, and acute kidney injury after ingesting approximately 30 tablets of lamotrigine (6000 mg) and fluoxetine (600 mg). Her recovery was further complicated by dysphagia and vocal cord dysfunction. This report highlights the importance of prompt and intensive supportive care and draws attention to the potential for uncommon cranial nerve involvement following severe mixed‑drug intoxication.

## Introduction

Lamotrigine is a phenyl‑triazine derivative, a second-generation antiepileptic commonly used as an anticonvulsant and mood stabilizer [1], while fluoxetine acts as a selective serotonin reuptake inhibitor. Both medications are frequently co‑prescribed in individuals with bipolar disorder who also experience anxiety‑related symptoms. Combination therapy is often considered in cases of treatment‑resistant bipolar depression [2], with the intention of improving therapeutic synergy while maintaining tolerability.

Although generally well tolerated, overdose of either drug can produce significant neurological and systemic toxicity [3], sometimes mimicking acute neurological or cardiac emergencies. Neurotoxicity may be masquerading as meningoencephalitis or drug-induced cardiotoxicity conditions that may progress to malignant arrhythmias or cardiogenic shock, complicating timely diagnosis and management. Classic manifestations of lamotrigine toxicity include dizziness, nystagmus, and gait instability; however, there are case reports suggestive of Stevens-Johnson syndrome and toxic epidermal necrolysis after initiation of therapeutic doses of lamotrigine. However, massive ingestion, particularly when combined with other psychotropic agents like fluoxetine, has been associated with more severe outcomes, including status epilepticus, coma, multiorgan dysfunction, features of serotonin excess [4], and enhanced sodium‑channel-mediated cardiotoxicity.

We describe the case of a 28‑year‑old woman with bipolar disorder who developed status epilepticus, atrial fibrillation, aspiration pneumonia, and acute kidney injury after ingesting approximately 30 tablets of lamotrigine (6000 mg) and fluoxetine (600 mg). Her recovery was further complicated by dysphagia and vocal cord dysfunction. This report emphasizes the role of early, aggressive supportive management in reducing morbidity and mortality from drug overdose and draws attention to possible cranial nerve involvement in severe mixed‑drug intoxication.

## Case presentation

A 28‑year‑old woman with a known diagnosis of bipolar disorder was brought to the Emergency Department (ED) by local police after being discovered unconscious in her home. The neighbor reported that, prior to being found, she had exhibited abnormal behavior characterized by slurred speech, unsteady gait, and nystagmus. They had also witnessed at least two episodes of generalized tonic-clonic seizures accompanied by bleeding from the mouth. It was later learned that she had recently experienced significant emotional stress following the death of her father one month earlier.

On arrival to the ED (Day 1), her Glasgow Coma Scale score was 9/15. Vital signs showed tachycardia at 122 beats per minute and blood pressure of 122/33 mmHg. Physical examination revealed multiple superficial bruises over her extremities, likely related to seizure‑associated falls. A urine pregnancy test was negative. Her regular medications included lamotrigine 200 mg once daily and fluoxetine 20 mg once daily. She was febrile, with a temperature of 102.4°F.

Initially admitted to the Neurology ward, she experienced rapid deterioration with two additional generalized tonic-clonic seizures associated with upward gaze deviation. She was administered intravenous midazolam and phenytoin sodium. Her neurological status subsequently worsened to E1V1M1, and she suffered an aspiration event, necessitating urgent intubation and transfer to the Critical Care Unit (CCU) after a few hours of admission.

On CCU admission (Day 1), her heart rate had risen to 155 beats per minute, and she remained febrile at 102°F, with maintained oxygen saturation (99%) on mechanical ventilation. Differential diagnoses considered were drug‑induced hyperthermia, status epilepticus‑related thermogenesis, central fever, aspiration pneumonia, and systemic infection, which was considered and ruled out after investigation.

An ECG demonstrated atrial fibrillation, for which she received intravenous amiodarone. Initial arterial blood gas (ABG) analysis (Table 1) showed mixed acidosis (severe lactic acidosis along with respiratory acidosis). Repeat ABG one hour later demonstrated significant improvement following ventilation, seizure control, and fluid therapy.

**Table 1:** Serial ABG (arterial blood gas) report

Parameters	Day One	Day Five	Day Eight	Reference Range	Unit
pH	6.966	7.286	7.528	7.35-7.45	-
pO₂	187	78.6	144	80-100	mmHg
pCO₂	78.2	38.7	25.2	35-45	mmHg
Na	143	134	138	135-145	mmol/L
K+	3.9	3.9	2.9	3.5-5.5	mmol/L
Cl	113	106	111	95-108	mmol/L
HCO₃	18.65	18.1	23.4	22-26	mmol/L
Anion Gap	12	10	4	8-12	mmol/L
Lactate	10.4	2.1	0.9	0.5-1.5	mmol/L

On Day 2 in CCU, further history obtained from her brother clarified the etiology: she had ingested approximately 30 tablets each of lamotrigine 200 mg and fluoxetine 20 mg, confirmed by empty blister strips found at home. Despite being on a midazolam infusion and phenytoin sodium, she continued to experience seizure episodes, leading to the addition of intravenous lacosamide.

On Day 3 in CCU, seizure recurrence persisted during attempts to taper midazolam, despite a triple‑antiepileptic regimen; hence, intravenous levetiracetam was introduced. Once status epilepticus had resolved, midazolam was gradually weaned. Her respiratory status improved as the aspiration pneumonia began to settle, allowing progressive reduction in ventilatory support.

Her management in CCU included mechanical ventilation, intravenous antiepileptic therapy, antibiotics, antiarrhythmic, antipyretic, stress ulcer prophylaxis, judicious fluid administration, venous thromboembolism prophylaxis, and nutritional support.

Her laboratory investigations revealed severe anemia, mild transaminitis, and a raised C-reactive protein. Serial laboratory findings are presented in Table 2.

**Table 2:** Laboratory findings on CCU days with reference range CCU – Critical care unit; WBC – White blood cell count; Hb – Hemoglobin; Neut – Neutrophil; AST – Aspartate transaminase; ALT – Alanine transaminase; Creat – Creatinine; BUN – Blood urea nitrogen; Alb – Albumin; CRP – C-reactive protein; T. Bil – Total bilirubin

Parameters	Day One	Day Five	Day Eight	Reference Range	Unit
WBC	10800	9620	11560	4.0-11.0	×10^3^ /µL
Hb	6.0	6.6	8.6	13.0-17.0	g/dL
Neut	84	73	89	60-70	%
Platelet	141000	161000	207000	4.5-5.5	cells/cmm
AST	145.90	114	45.50	0-35	U/L
ALT	59.40	54.4	50.60	0-45	U/L
BUN	26	21	18	6-21	mg/dL
Create	0.67	0.54	0.9	0.5-1.5	mg/dL
Alb	3.07	3.62	3.8	3.5-5.2	gm/dL
CRP	4.44	3.4	3.8	0.08-0.79	mg/dL
T. Bil.	1.3	1.84	1.9	0.2-1.0	mg/dL

Neuroimaging with MRI brain (with gadolinium) and cerebrospinal fluid analysis were unremarkable, effectively excluding meningoencephalitis. A two‑dimensional echocardiogram (LVEF 60%) and abdominal ultrasonography showed no abnormalities.

She was successfully extubated on Day 7 and transitioned to non‑invasive ventilation. By Day 9, she was stable enough to be transferred back to the Neurology ward.

During her ward stay, she developed dysphagia and dysphonia. Evaluation done by an otolaryngologist, including flexible endoscopy, identified vocal cord dysfunction. Vocal cord dysfunction was treated with conservative medical treatment, including laryngeal rest and voice therapy. Psychiatric evaluation was conducted, and her psychotropic regimen was revised to include fluoxetine, clonazepam, and sodium valproate. After six days of continued ward care, her fever resolved, seizures remained controlled, and she was discharged home in stable condition.

## Discussion

This case highlights the crucial importance of timely, intensive supportive management in patients presenting with severe central nervous system toxicity due to multidrug overdose. In particular, it demonstrates the complexities involved in treating refractory status epilepticus precipitated by the combined ingestion of lamotrigine and fluoxetine.

Previous literature indicates that seizures occur in approximately 10-20% of patients with significant lamotrigine overdose [5], particularly when presenting with altered consciousness. These episodes, especially when refractory, are considered medical emergencies given their association with unfavorable neurological outcomes and the need for rapid escalation of therapy. In our patient, conventional measures such as benzodiazepines and phenytoin sodium were insufficient, requiring titrated midazolam infusion and the subsequent addition of lacosamide and levetiracetam. This approach aligns with established treatment algorithms for status epilepticus [6], which emphasize early airway protection and aggressive uptitration of anticonvulsant therapy when initial agents fail.

Lamotrigine's mechanism primarily involves voltage‑gated sodium channel inhibition, reducing neuronal excitability and glutamate release. While therapeutic dosages are typically well tolerated, excessive serum concentrations may lead to off‑target sodium channel blockade in cardiac tissue. This may manifest clinically as conduction delays, arrhythmias, and hemodynamic compromise. Neurologically, toxicity may present with cerebellar dysfunction, persistent seizures, or deep coma. Co‑ingestion with fluoxetine [7], as seen in this case, can potentiate refractory seizures and neurotoxicity.

Lamotrigine is predominantly metabolized by glucuronidation, with an elimination half-life of 24-35 hours, potentially extending to around 60 hours in scenarios of hepatic impairment or concomitant medications. Fluoxetine, metabolized to norfluoxetine via hepatic demethylation, has an elimination half‑life of 48-72 hours [8], with its active metabolite persisting for 7-9 days. Both drugs exhibit non‑linear pharmacokinetics [9] during overdose due to metabolic pathway saturation, which may significantly extend their elimination and intensify toxic effects. This case's refractory status persisted until Day 4, prompting the addition of four antiepileptic drugs for seizure control. It underscores the value of careful dose adjustments of psychotropic drugs, and whenever available, therapeutic drug monitoring should be done, though such resources are often limited in developing healthcare settings.

Massive lamotrigine ingestion (typically >4,000 mg) frequently necessitates rapid escalation of benzodiazepine therapy [10] and early introduction of multiple antiepileptic agents. Interestingly, Alyahya et al. [11] reported that serum drug concentration, rather than estimated ingested dose, correlates more reliably with toxicity severity. Fatal outcomes described by French et al. [12] and Nogar et al. [13] following ingestion of 4,000 mg and 7,500 mg, respectively, further reinforce the concept of concentration‑dependent toxicity. In both reports, profound neurological and cardiac toxicity, including refractory seizures, conduction abnormalities, and cardiorespiratory collapse, were prominent features. Our case shares several parallels, including severe status epilepticus, aspiration pneumonia, atrial fibrillation, and sepsis, although ultimately with a favorable outcome.

Renal dysfunction, as observed in our patient, may further impair lamotrigine clearance indirectly via metabolite accumulation. Additionally, fluoxetine co‑ingestion has been implicated in amplifying both neurological and cardiac manifestations. This has been supported by reports such as that of Chistyakova et al. [14], who described delirium and neurotoxicity following combined ingestion of these agents.

Currently, no standardized treatment protocol exists for lamotrigine overdose. Most published cases emphasize timely gastric decontamination with activated charcoal (where feasible) and comprehensive supportive measures, including airway management and circulatory stabilization. Hemodialysis and hemoperfusion [15] are not routinely recommended due to lamotrigine’s high protein binding, though they may be considered in rare, extreme cases with persistent clinical deterioration despite maximal supportive therapy.

In our patient, the clinical picture was further complicated by aspiration pneumonia, transient atrial fibrillation, and later, vocal cord dysfunction. Her cardiac arrhythmia responded to amiodarone therapy. Aspiration-related lung injury improved with ventilatory support and antimicrobial therapy. The vocal cord dysfunction [16], which manifested as dysphagia and dysphonia, likely reflected intubation-associated trauma such as recurrent laryngeal nerve neuropraxia, though systemic toxic neuropathy cannot be fully excluded. The rapid symptomatic improvement following conservative management, including swallowing rehabilitation and laryngeal rest, favored an intubation-related etiology.

Overall, this case underscores the need for preparedness for advanced critical care interventions, including high‑dose sedative infusions, early intubation, and multi‑agent antiepileptic therapy when managing severe lamotrigine and fluoxetine toxicity. Prompt recognition and comprehensive supportive care remain the cornerstone of improving outcomes in such complex overdoses.

## Conclusions

Severe toxicity from lamotrigine and fluoxetine ingestion constitutes a multisystem medical emergency that demands rapid recognition and comprehensive supportive management. This case illustrates the successful stabilization of a young woman who developed profound neurological impairment and refractory status epilepticus after consuming large quantities of both medications. Beyond the expected sodium channel-related conduction delays, clinicians must remain vigilant for atypical cardiac manifestations, such as atrial fibrillation, as well as uncommon neurological complications, including transient vocal cord dysfunction.

In the absence of a targeted antidote, optimal outcomes depend on meticulous attention to airway protection, seizure suppression, cardiovascular monitoring, renal support, and early treatment of secondary complications such as aspiration and infection. A coordinated, multidisciplinary approach involving intensive care, toxicology, cardiology, neurology, otolaryngology, speech therapy, and psychiatric services is essential not only for acute stabilization but also for long‑term risk mitigation and prevention of recurrence.
